# Account of an Amputation Performed with Success in the Middle of the Foot

**Published:** 1788

**Authors:** T. Turner

**Affiliations:** jun. Surgeon at Yarmouth, in Norfolk


V. *Account of an Amputation performed with Succefs in the Middle of the Foot. Communicated in a Letter to Dr.* Simmons, *F. R. S. by Mr.* T. Turner, *jun. Surgeon at Yarmouth, in Norfolk.*

AS amputation in the middle of the foot is an operation very rarely practicable, and I do not know that there is an account of fuch an operation in any furgical publication, I take the liberty, Sir, of fending you the following cafe, to be inferted, if you think fit, in the London Medical Journal.

Mary Sloman, aged twenty-three years, had a confiderable tumour upon the top of her foot. It began about two years fince by a fmall and and immoveable ſwelling upon the metatarſal bone that ſupports the ſecond toe, near to the joint of the toe, and gradually increaſed in ſize till its dimenſions were as follows, viz. from the inner to the outer ſide it meaſured four inches and a half; from the anterior to the poſterior part its dimenſions were the ſame; and from the center of the upper part of the tumour to the ſole of the foot, between the great toe and the next, it meaſured three inches.

The tumour was of a ſtony hardneſs, without fluctuation in any part, and immoveable. It was ſituated upon, and firmly attached to the metatarſal bones of all the toes, the little one excepted. It adhered alſo to the firſt joint of each of thoſe toes, and to the ſubjacent parts; and it had ſeparated the great toe from that next to it conſiderably.

The tumour reached about half way up the metatarſus, and was very painful. The foot was entirely uſeleſs.

In conſultation it was thought hard for the patient to loſe her leg at the uſual place below the knee, and it was agreed that as much of the foot ſhould be ſaved as could be after completely removing the tumour and the parts connected with it.

The

The operation was performed, in the preſence of the gentlemen who met at the conſultation, on the 8th of October laſt, by the double inciſion; and in order to ſave as much of the integuments as poſſible, a ſmall part of them, covering the tumour, was included in the firſt inciſion. As the integuments retracted very inconſiderably, it was not poſſible to preſerve ſo much of them as was intended. The foot was ſawn through at the ſuperior part of the metatarſus, and the hæmorrhage was eaſily ſuppreſſed. The ſtump was completely cicatriſed on the 18th of December, without the ſmalleſt exfoliation or trouble whatever.

The nature of the tumour was not known till after the operation, when it was diſſected and found to be a true ſchirrus. The bones of the foot were not at all affected.

The idea of operating in the middle of the foot was ſuggeſted by Dr. Aikin, of this town; and he has the merit of all the advantages the woman will certainly derive from the operation's having been performed at that part.

*Yarmouth, Norfolk,*

Dec. 26th, 1787.

